# PReS-FINAL-2130-A: Effectiveness of intravenous cyclophosphamide in severe or refractory juvenile dermatomyositis - a national cohort study UK and Ireland

**DOI:** 10.1186/1546-0096-11-S2-P143

**Published:** 2013-12-05

**Authors:** E Moraitis, K Arnold, LR Wedderburn, CA Pilkington

**Affiliations:** 1 Rheumatology, Great Ormond Street, London, UK; 2 Rheumatology, UCL, Institute of Child Health, London, UK

## Introduction

Evidence suggests that early and aggressive treatment in Juvenile Dermatomyositis (JDM) improves outcome and prevents complications. Cyclophosphamide has been used as a second-line agent in the treatment of severe or refractory JDM. Published data on the effectiveness of cyclophosphamide in JDM are limited to a previous small case series and case reports.

## Objectives

To describe the response to, and evaluate the effectiveness of, intravenous cyclophosphamide in the patients with JDM from the UK JDM National (UK and Ireland) Cohort and Biomarker Study and Repository for Idiopathic Inflammatory Myopathies.

## Methods

The JDM National (UK and Ireland) Cohort and Biomarker Study and Repository for Idiopathic Inflammatory Myopathies (n = 410) prospectively collects clinical and laboratory data and samples on all children recruited to the study, using standardised protocols. 56 patients in the cohort were treated with cyclophosphamide between 2000-2011. Eight patients were excluded due to incomplete data or short follow up. The remaining 48 had a diagnosis of definite JDM, probable JDM or JDM overlap (Bohan and Peter criteria), with a minimum of 12 months follow up after the first dose of the cyclophosphamide. Demographic data, core set measures of disease activity, skin data, laboratory measures, treatment data were analysed at baseline, 6, 12, 18, 24 months and last follow up.

## Results

Indications for starting the cyclophosphamide were ulcerative or severe skin disease, profound muscle weakness, lung disease, gastro-intestinal vasculopathy or refractory disease. All patients starting with muscle weakness (n = 44) significantly improved at 12 months, and the gains were maintained at follow up. Physician VAS was available at baseline for 32 patients and these all improved by 12 and 24 months, and for 31 remained stable at follow up. At last follow up, 26/46 (56%) had no rash, 32/46 (69%) had normal nailfolds, 37/45 (82%) had no Gottron's, and calcinosis has resolved in 9/14 (64%). The steroid dose was decreased by 65.8% at 6 months, and the majority of patients were off steroids or on very low doses at 24 months. Patients receiving cyclophosphamide had a significant decrease in steroid dose by 6 months, with most of the patients coming off steroids between 18 and 24 months.

## Conclusion

These data suggest that cyclophosphamide provides clinical benefit in JDM patients with severe or refractory disease, improving both muscle and skin domains.

## Disclosure of interest

None declared.

